# Supplementary figures and images for: The Growth-Inhibitory Effect of Increased Planting Density Can Be Reduced by Abscisic Acid-Degrading Bacteria
Source: Biomolecules. 2023 Nov 19;13(11):1668. doi: 10.3390/biom13111668 (PMC10669761; doi:10.3390/biom13111668)

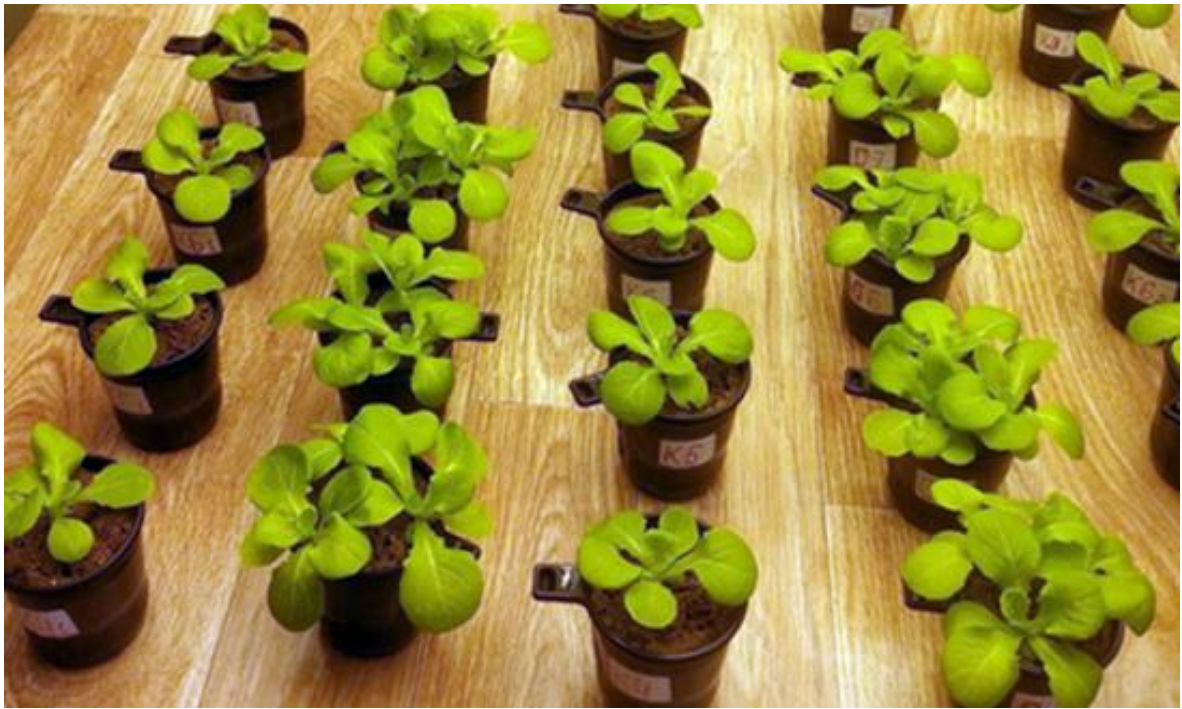

**Non-inoculated**

**Inoculated** with  
*P. Plecoglossicida* 2.4-D

Supplement: Supplementary file 1 [file biomolecules-13-01668-s001.zip › biomolecules-2694063-supplementary.pdf]
